# Experimental Study on the Modular Vertical Greening Shading in Summer

**DOI:** 10.3390/ijerph191811648

**Published:** 2022-09-15

**Authors:** Shenglin Bao, Simin Zou, Mingqiao Zhao, Qiuyu Chen, Baofeng Li

**Affiliations:** 1 School of Architecture & Urban Planning, Huazhong University of Science and Technology, Wuhan 430074, China; 2 Hubei Engineering and Technology Research Center of Urbanization, Wuhan 430074, China; 3 School of Civil Engineering, Central South University, Changsha 410075, China; 4 School of Architecture and Art, Central South University, Changsha 410075, China

**Keywords:** plant shading, modular vertical greening, cooling effect, CO_2_ reduction

## Abstract

Previous studies have shown that vertical greening has a significant cooling and energy-saving effect, most of which are applied to opaque walls. However, windows are the critical factor contributing to the indoor thermal environment. This study developed a modular vertical greening shading device (MVGSD), and introduces its detailed structure: water supply mode, plant selection, and substrate preparation. To investigate the thermal performance of MVGSD, a structural model test was carried out. The results show that MVGSD has a noticeable effect on indoor temperature. Specifically, the greatest indoor temperature can be reduced by 4 °C and effectively low the concentration of CO_2_ (The CO_2_ absorption rate is 53.1%). In addition, the characteristics of the louver shading and MVGSD were compared, and it was found that the indoor temperature by using MVGSD is 2.6 °C lower than the louver. It is also worth mentioning that indoor humidity is improved by MVGSD, which has a beneficial effect on the thermal comfort of human beings.

## 1. Introduction

Buildings consume approximately 30–40% of the world’s energy [1]. The heat exchange between the building envelope and the outdoors caused by heating and air conditioning energy consumption accounts for 56–58% of the total building energy consumption. The windows are the weakest part of energy efficiency in the four main building envelopes(windows, doors, walls, roofs, and floors) [2], thus regarded as potential to save energy. Windows usually cause overheating and dazzle in the summer due to excessive solar radiation penetration. Compared to walls and floors, the thermal conductivity and air infiltration coefficients of windows are higher, which results in increased energy consumption for cooling air conditioners in summer. According to tests by the researchers, if the windows of southern rooms can avoid sunlight entering the room from 10:00 to 14:00 in summer, the indoor temperature can be reduced by an average of 2–5 °C during this period, reducing building operating costs by around 30% [3]. Shading devices, as a passive strategy, have a significant effect on the energy efficiency of windows and have an essential impact on the energy efficiency of the transparent building envelope.

**Figure 1:**
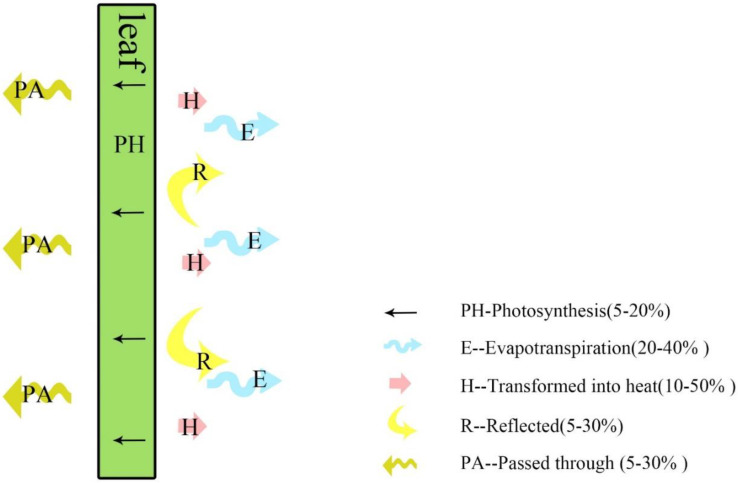
Plant leaf heat balance analysis.

Many studies have shown that vertical greening has a cooling and energy-saving effect [4,5,6,7,8,9]. Vertical greening reduces the ambient temperature around the plant canopy through the absorption of solar radiation, transpiration by the plant leaves, and evaporation of water from the substrate. At the same time, the planting substrate and the plant canopy also have a certain thermal insulation effect. The plant canopy intercepts the solar radiation reaching the surface of the buildings since the shading mechanism of plants and shading devices is different, converts solar energy into bioenergy through photosynthesis, absorbs 5–20% of solar radiation, and reflects 5–30% of solar radiation, and transpiration with 20–40% of solar radiation [10] (Figure 1). The transpiration of leaves absorbs heat, therefore, the plant temperature did not increase significantly [11]. When the energy lost by radiation and transpiration is greater than the amount of solar energy absorbed by the leaf, the leaf temperature becomes lower than the ambient temperature [12]. However, the temperature of the sunshades will increase significantly after absorbing solar energy, and part of the heat will be transferred to the room through radiation, so the shading effect of plants is better in summer. In recent overviews of the development of vertical greenery systems [6,7,8,13,14,15], vertical greenery systems are divided into two main categories: living walls and green facades. By comparing the thermal reaction mechanisms, green facades only rely on the plant canopy to block solar radiation, while living walls include planting substrate, plant canopy, and other artificial structures, which can block solar radiation and reduce the nearby air temperature. It was found that the indoor temperature of living walls is 3~4 °C lower than that of green facades [16]. Also, the energy-saving efficiency of living walls is 15.1% higher than that of climbing plants in Spain [17].

Most of the current research on the cooling and energy saving of vertical greening mainly focuses on the opaque enclosure (roofs and walls), and only a few studies have discussed the thermal environment impact on the windows. In the Netherlands, Stec planted climbing plants inside the double glazing and found that climbing plants reduced indoor temperatures twice as much as blinds under the same solar radiation conditions [18]. In addition, the plants stayed below 35 °C when the temperature of the blinds approached 55 °C. The climb plants saved approximately 20% in cooling energy. Wang added Tillandsia usneoides in the double glass façade and found that the surface temperature of the inner façade was significantly reduced [19]. In Thailand, Sunakorn used climbing plants to make a ‘bio-facade’ for shading and reduced the indoor air temperature by up to 9.93 °C [20]. Kenneth planted a local creeper outside a building window and the research results showed that the temperature dropped by 4~6 °C under the highest temperature in summer [21]. Zheng designed a portable green window shading device using three climbing plants and found that the shading coefficient was 0.28 at 80% leaf cover, and the cooling energy and heat flux were reduced by 11.5% and 64.8%, respectively [22].

Previous research mainly applied modular vertical greening to walls. For plants on windows, climbing plants are mostly used, using the plants as the second facade outside the window. Very little research has been conducted on applying modular vertical greening to window shading and its thermal effect on shading in transparent enclosures. Due to the limitation of growth height and uncontrollable growth directions, the climbing plants lead to the instability of the shading area and are unsuitable for shading windows in high-rise buildings. However, the energy consumption of facilities in China is mainly concentrated in high-rise buildings. Modular vertical greening can adapt to transparent enclosure structures of different sizes, densities, angles, and heights. Therefore, it is necessary to conduct more research on modular vertical greening shading for transparent envelope structures in high-rise buildings to fill this research gap. This paper proposes the application of modular vertical greening to window shading—a modular vertical greening shading device (MVGSD)—and investigates the cooling effect of MVGSD on windows through two sets of comparative experiments. The thermal parameters of rooms installed with MVGSD and louvers in China’s hot summer and cold winter areas are measured and the thermal performance differences between the two shading components are compared.

## 2. Materials and Methods

### 2.1. Design of the MVGSD

**Figure 2:**
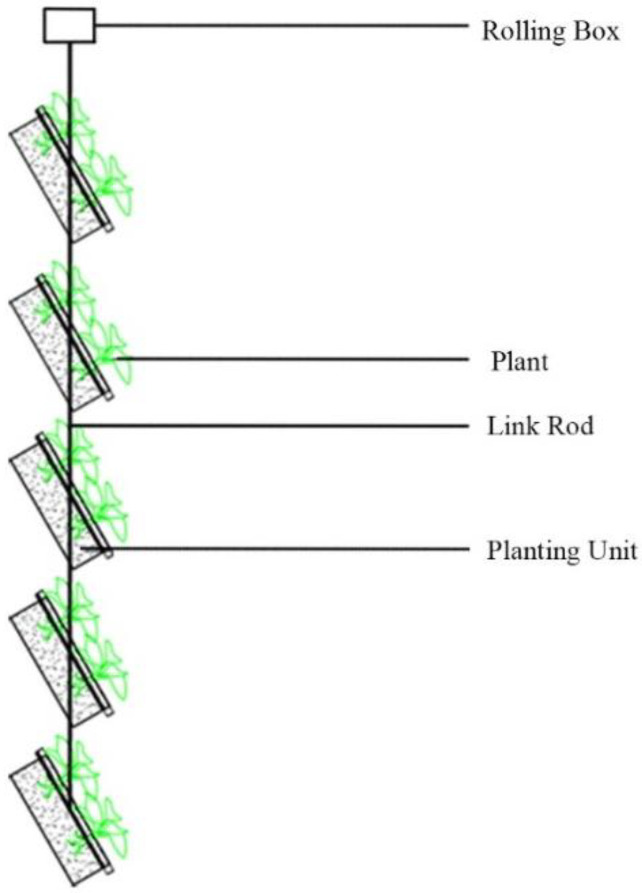
Simplified shading model of MVGSD.

Due to the diversity of window sizes and forms, modular planting units are adopted, which can be freely matched and combined according to different buildings. At the same time, they are also conducive to easy installation, disassembly, and replacement, which is a simple operation and can be produced massively. Considering that the MVGSD needs to be adjusted according to solar radiation, the design refers to the traditional rotation of the louvers (Figure 2).

#### 2.1.1. Water Supply

Water supply is the most routine maintenance of vertical greening to ensure the typical growth of plants. Considering that the MVGSD needs to be installed at different heights, the watering frequency should be reduced as much as possible. Therefore, the MVGSD adopts the “trace irrigation” method, which has been widely used in agriculture to actively supply water according to the needs of plants, which can save water efficiently. At the same time, no pressure device is required for trace irrigation to promote the movement of water molecules. Plants lose water with transpiration, while the water is transported to the soil through the capillary and absorbed by plant roots. When the soil reaches saturation, the water supply stops and the whole process of autonomy does not consume energy. Under the capillary, water is actively absorbed into plant roots and soil, the whole irrigation process is carried out in the soil. Compared with traditional irrigation, the evaporation of water is significantly reduced.

#### 2.1.2. Plant Selection

Considering the particularity of the location of MVGSD, the following aspects should be regarded in plant selection:

- Resistant to high temperature. In summer, the average temperature in Wuhan is above 30 °C, and some extreme temperatures will reach 40 °C. The physical environment in which the plants are located may be higher than the indoor temperature, and the water evaporation is fast. The plants need to maintain vitality in harsh environments and have a specific resistance to high temperatures.
- Short and well-developed root systems. The size of the planting unit is restricted due to the relatively limited space in the window, which requires plants with short roots that can grow steadily in a smaller area.
- Lower canopies and light. As plants are planted inside windows, the shielding effect of plants should be minimized, so the plants with a lower canopy should be selected. To reduce the weight of the whole installation, the plants also need to be as light as possible.
- Moderate growth rate. Plants growing too fast would lead to more burden on the load-bearing components, increase the maintenance cost later, and more operations for management and care.

The ideal plant is determined as a perennial herb based on the above basic principles of plant selection. Considering the climate conditions, plant cost, and tolerance, Ophiopogon japonicas is selected as the plant of MVGSD.

#### 2.1.3. Planting Substrate

Planting substrate is the basis of plant survival and growth. Compared with horizontal planting, the living conditions of facade plants are relatively unfavorable. Thus, the water retention, stability and lightness of the MVGSD substrate should be considered. The pH value should be 5.5~7.0, the nutrients should be moderate, and the cost of the cultivation medium should be reduced to the greatest extent. A literature search compared the dry bulk density, thermal conductivity, PH, porosity, and conductivity of the commonly used planting substrates, and three basic matrixes, nutrient soil, coir, and perlite, were selected. Nine planting matrixes with different proportions were designed through an orthogonal experiment (Table 1). In order to ensure the accuracy of measurement, three measuring points are selected for each substrate to measure its PH and electrical conductivity, and the average value is taken as the final comparison data (Table 2).

**Figure 3:**
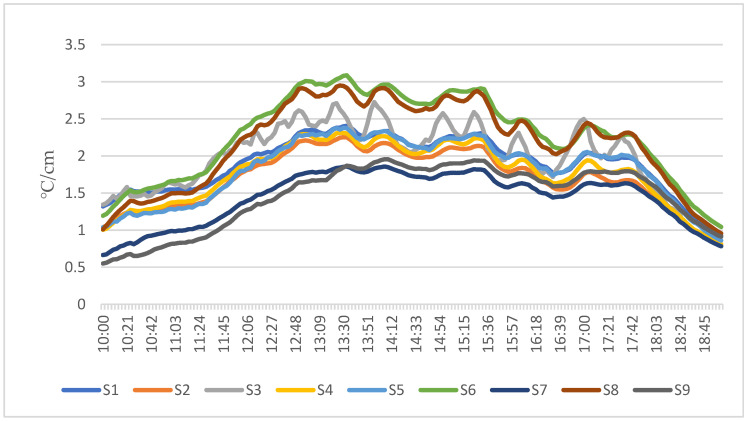
Temperature difference per unit thickness of the substrate.

Water content is an essential factor affecting the soil’s thermal conductivity. As the water content in the soil is constantly adjusted according to the changes in solar radiation, the thermal conductivity of the substrate also changes. In order to measure the actual heat transfer changes as much as possible and select the best substrate, the soil temperature of 9 mixed substrates under sunny conditions was measured with the help of a heat flux inspection instrument. The temperature variation of the bottom of the 9 substrates was compared with the same amount of solar radiation obtained. The temperature variation per unit thickness was calculated for the 9 substrates (Figure 3).

The S6 substrate was selected as the planting substrate for the MVGSD by comparing the nine substrates in terms of weight, porosity, PH, water content under capillary action, thermal conductivity and EC (Table 3).

### 2.2. Design of the Experiment

#### 2.2.1. Experimental Settings

**Figure 4:**
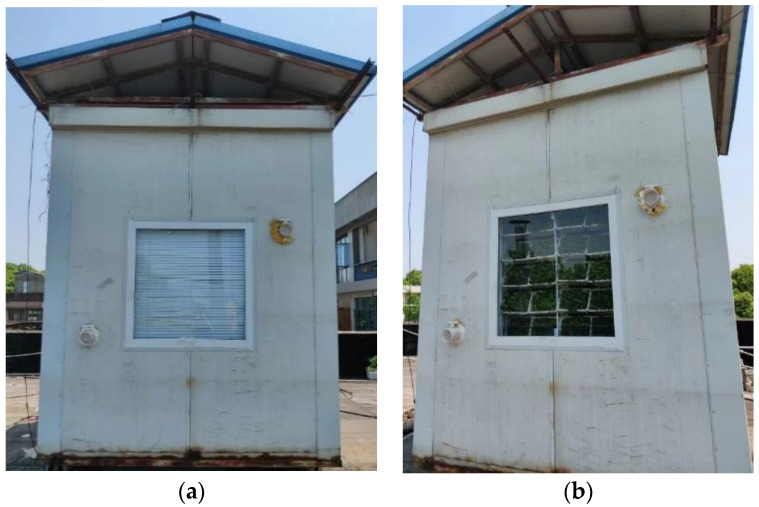
Experimental room: (**a**) Window with louver; (**b**) window with MVGSD.

In order to study the impact of MVGSD on the indoor thermal environment, two groups of comparative experiments were conducted in two identical experimental rooms on the roof of a building in Wuhan (Table 4). The size of the two experimental rooms was 2.3 m × 2.6 m × 2.8 m with a window (1 m × 1 m) (Figure 4). As the cooling effect of vertical greening in the west is the best [23], so the experiments were carried out in the west direction. There are no buildings or objects west of the two rooms, and they do not block each other.

#### 2.2.2. Equipment and Layout

**Figure 5:**
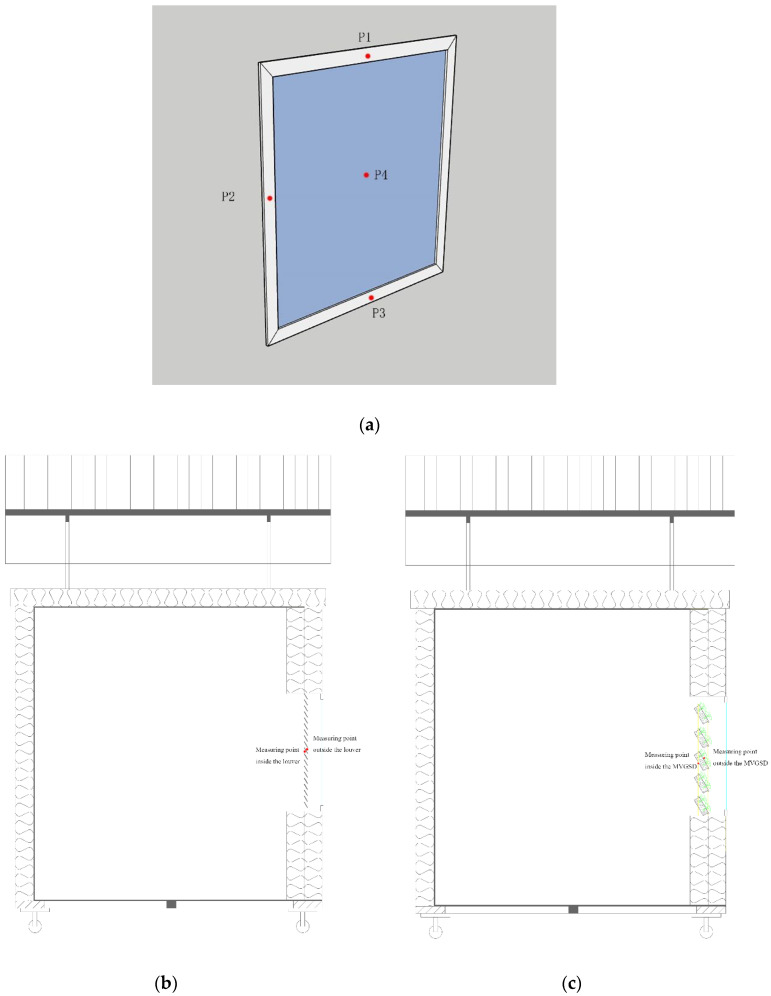
Measuring points of windows (**a**), louver (**b**), and MVGSD (**c**).

To study the impact of MVGSD on the indoor thermal environment and CO_2_ concentration. The instruments include a temperature and humidity recorder, a thermal conductivity coefficient instrument and meteorological station, and a CO_2_ recorder (Table 5). A temperature and humidity recorder record the indoor temperature and relative humidity. The thermal conductivity coefficient instrument measure the temperature of the inner surface of the window (four measuring points), the temperature of the louver and the MVGSD. Weather monitors record the outdoor temperature, humidity and solar radiation. The CO_2_ recorder records the indoor CO_2_ concentration. In order to study the different heat transfer reactions between the two kinds of sunshade components, temperature measuring points were installed on both sides of the two kinds of sunshades and the inner side of the window (Figure 5).

**Table 5:** List of experimental instruments.

Instrument Name	Picture	Detailed Parameters
Temperature and humidity recorder	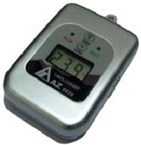	AZ Instrument (model 8829); the humidity measurement error is ±3% when the temperature is 25 °C and the humidity is 10~90%, and the error is ±5% in other environments.
Thermal conductivity coefficient instrument	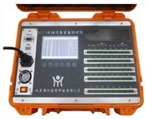	Operating temperature range of the instrument: 0-50 °C, storage temperature range: −20~60 °C, operating humidity range: less than 85% RH
Weather monitor	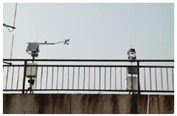	Model: 2012 vantage pro2 Parameters: air temperature: measurement range −40~65 °C, resolution 0.1 °C, accuracy ±0.5 °C; Relative humidity: 1~100%, resolution 1%, accuracy ±3–4%; Solar radiation: 0~1800 w/m^2^, resolution 1 w/m^2^, accuracy ±5%;
Hobo MX CO_2_ recorder	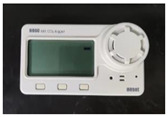	Measurement range: 0~5000 ppm Accuracy: ±50 ppm at 25 °C and less than 90% RH

## 3. Results

### 3.1. Experiment 1: Comparison between the MVGSD and the Unshaded Window

**Figure 6:**
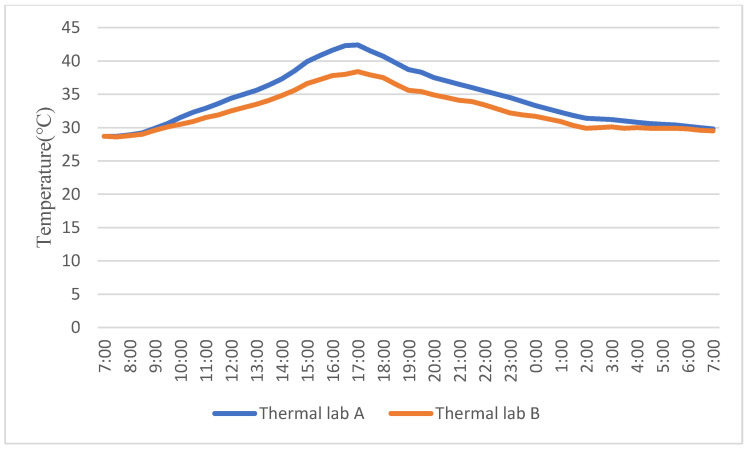
Experiment 1: Indoor temperature comparison.

In order to compare the effects of green window shading components on the indoor temperature and CO_2_ concentration, one thermal lab installed with an MVGSD and another with an unshaded window were monitored. There is no air conditioning in the experimental room and windows were closed to minimize the room’s heat. It was found that the MVGSD had a significant cooling effect during the day, especially from 16:30 to 17:00, when the temperature in room A reached 42.4 °C, while room B was only 38.4 °C. Overall, the average temperature of room B was 1.8 °C lower than A (Figure 6).

**Figure 7:**
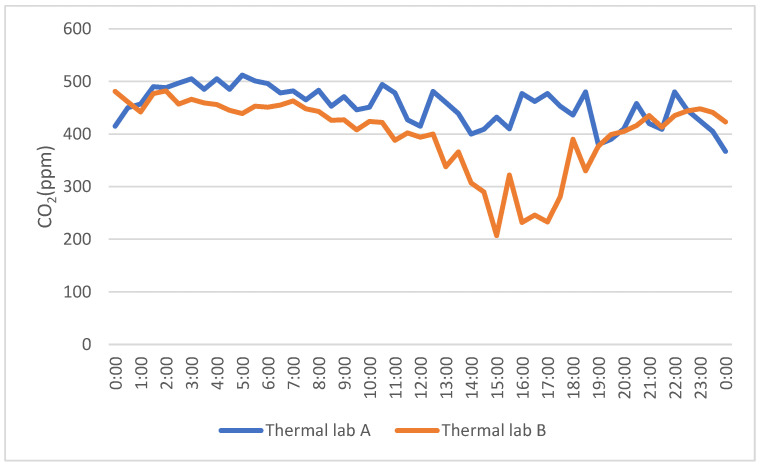
Experiment 1: Indoor CO_2_ concentration comparison.

From the perspective of indoor CO_2_ concentration, the difference was only 28 ppm and was not significant at the beginning. After 8:00, the difference gradually increased due to the photosynthesis of plants. At 15:00, the CO_2_ concentration in Room B was the lowest, only 207 ppm (Table 6), the initial concentration of 46.9%, and the CO_2_ absorption rate was 53.1%. At 16:00, maximum difference occurs (up to 245 ppm), the average CO_2_ concentration difference between the two rooms during the daytime (8:00–20:00) was 97.48 ppm. At night, the indoor CO_2_ concentration increased again caused by the respiration of plants, but it was 58 ppm lower than the indoor concentration at the beginning of the experiment (Figure 7).

### 3.2. Experiment 2: Comparison of Louver and MVGSD

**Figure 8:**
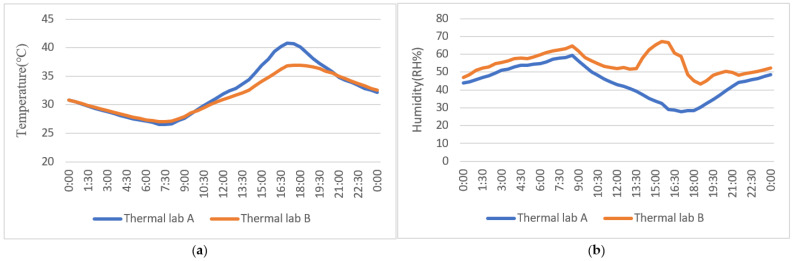
Experiment 2: (**a**) Indoor temperature comparison; (**b**) indoor humidity comparison.

The louver is a common sunshade component used in buildings. Experiments between the louver and the MVGSD were compared on 12 September 2021.As shown in Figure 8, the temperature difference between the two rooms in the morning was insignificant. Comparing the indoor temperature of the two rooms in the afternoon (12:00–18:00), the indoor temperature of B was 2.6 °C lower than that of A on average, and the maximum difference was 4 °C at 16:00 (Figure 8a). Due to the transpiration of the substrate and the leaves of the MVGSD, the indoor humidity also increased. Significantly at 13:30–16:00, the evaporation speed of the MVGSD accelerated under the action of solar radiation, resulting in rapidly growing humidity. The humidity in room B varied from 43.4% to 67.3% throughout the day (Table 7), and the humidity was relatively comfortable. However, the indoor humidity of A was 27.7–40% from 13:15 to 20:35, lower than the indoor relative humidity range of 40–70% recommended by the Chinese Design code [24]. The minimum indoor humidity in room A was 27.7%, and too dry to uncomfortable for the human body (Figure 8b).

**Figure 9:**
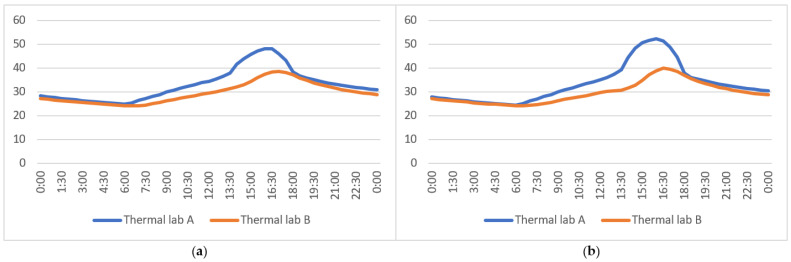
(**a**) Inside temperature of the sunshade; (**b**)outside temperature of the sunshade.

Compared to the internal and external temperature, the external surface temperature of the louver reached 52.21 °C at 16:00 and 48 °C on the backside. The maximum temperature on both two sides of the MVGSD was 39.79 °C and 38.55 °C and was 15.7 °C and 11.26 °C lower than the louver, respectively (Table 8). The outer temperature difference is more significant than the inner, indicating that the solar radiation reaching the substrate surface is less under the photosynthesis of plants. Furthermore, the substrate surface temperature rises slowly while the louver absorbs solar radiation, and the temperature rises rapidly (Figure 9). During the 8:00–20:00 period, the average temperature outside the MVGSD was 6.54 °C lower than that of the louver, and the average temperature inside the MVGSD was 5.43 °C lower than that of the louver.

**Figure 10:**
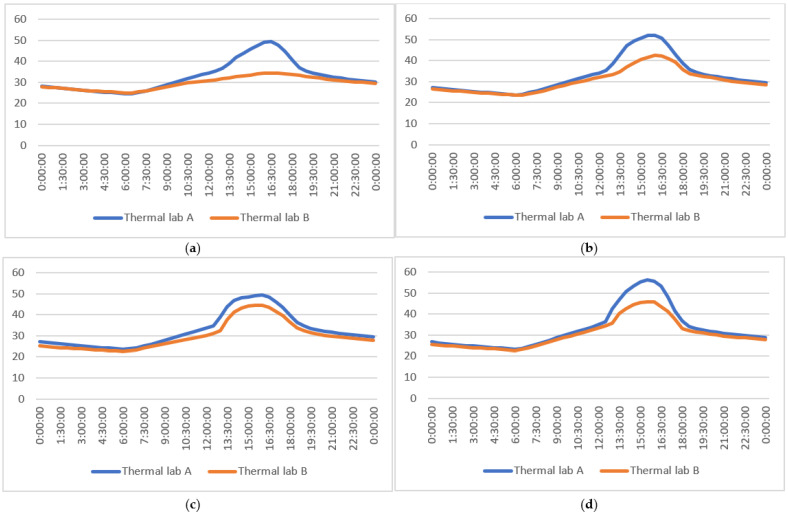
(**a**) P1 temperature; (**b**) P2 temperature; (**c**) P3 temperature; (**d**) P4 temperature.

In order to verify the difference in the heat transfer mode between the two forms of shading, the internal surface temperature of windows in the two rooms was compared. Figure 10 shows that the internal surface temperature of a window in room B is lower than that of room A. The maximum temperature difference between P1–P4 of a window in room B and room A was 14.93 °C, 10.28 °C, 6.45 °C, and 10.6 °C, respectively. The average temperature of the four measuring points on the inner surface of the window in room B was 3.19 °C, 2.46 °C, 2.5 °C, and 2.56 °C lower than that of room A (Table 9).

## 4. Discussion

### 4.1. Heat Gain by the Inner Surface of the Window

**Figure 11:**
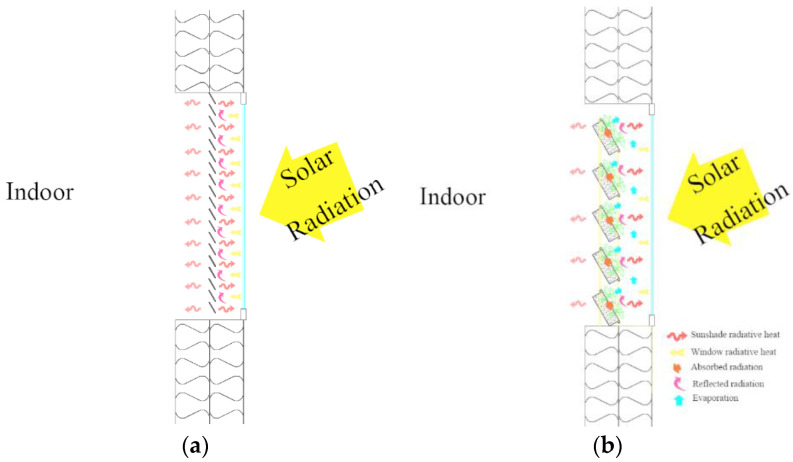
Louver (**a**) and MVGSD (**b**) shading heat transfer.

The heat of windows mainly comes from solar radiation, heat conduction of the air layer, and sunshades’ reflected radiation and radiative heat. Because of the same experimental location and conditions, the heat gain from solar radiation received by the outer surface of the window were essentially the same, and the latter three factors mainly caused the difference in temperature between the measured points P1–P4. As shown in Figure 11, the inner surface of the window in Thermal lab B receives only part of the solar radiation because of the plants’ photosynthesis and leaf characteristics, which absorb and reflect part of the solar radiation. In addition, the evaporation of water from the leaves and substrate absorbed heat from the “air layer”, which is lower than that of A and reduces the conductive heat of the “air layer” (between the window and the MVGSD). No significant temperature increase was observed in the leaves, and the surface temperature of the substrate was 15.7 °C lower than that of the louvers. The radiative heat (L) was proportional to the object’s temperature (Equation (1)), and the radiative heat of the MVGSD was much lower than that of the louver. By analyzing the difference between the four measuring points, it is found that the temperature difference between the two rooms shows a decreasing trend from top to bottom, with the largest temperature difference at the top. The maximum value of P1–P3 in lab A is similar, while the temperature difference of P1–P3 in lab B is 10 °C, which indicates that the MVGSD and windows form a microclimate, proving that the evaporation of water from the plants and substrate in the MVGSD absorbs heat from this “air layer” and changes the microclimate, which is a characteristic that the louvers do not have.

L = εσT^4^ (1)

where L is the thermal radiation, σ is the Stefan–Boltzmann constant with a value of 5.669 × 10^−8^ W/(m^2^K^4^), and ε is the emittance value of the surface. T is the thermodynamic temperature of the object surfaces, K.

### 4.2. Heat Gain by the Sunshade

The outer side of the sunshade receives heat mainly from solar radiation, radiant heat from the window and conductive heat from the “air layer” formed between the sunshade and the window, while the heat of the inner surface comes mainly from conductive heat from the indoor air and sunshade outer side. The solar radiation is the same, and the temperature difference is mainly due to the latter two factors. The louver transmits a part of the radiation to the interior, which increases the indoor and inside the louver temperature. While the other part reflected on the “air layer,” which increases the temperature and the conductive heat of the “air layer”.

**Figure 12:**
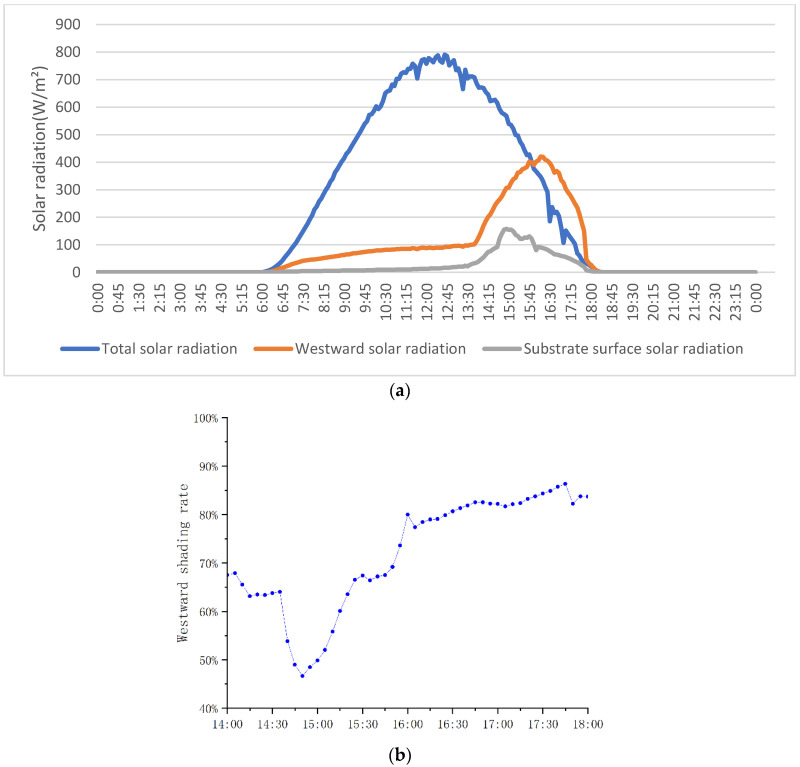
Solar radiation variation (**a**) and Shading rate of MVGSD (**b**).

Analyzing the heating outside MVGSD, it can be seen from Figure 12a that the western solar radiation increased rapidly from 13:40, but under the shelter of the plant canopy, the solar radiation reaching the substrate surface was significantly reduced, and the shading efficiency of leaves from 14:00 to 18:00 was 46.69–86.32%(Figure 12b), with an average shading rate of 71.98%. It is the main contributor to heat gain, and the plant substrate also intercepts the portion that passes through the leaves. In addition, the photosynthesis of plants also converts 5–20% of solar radiation into bioenergy to provide energy for the survival and growth of plants, which is also a feature that other artificial materials, such as shutters, do not have. Secondly, the low temperature on the inner side of the window (Figure 10) leads to a correspondingly low radiative heat to the MVGSD. Thirdly, the temperature of the air layer does not increase much under the evaporation of the MVGSD. It can be seen from Figure 7B that the indoor humidity reaches the maximum value at 16:00, which is also the peak value of the westward solar radiation. The results indicate that the evaporation of plants and substrates accelerates, increasing indoor humidity, and the evaporation of water absorbs much more heat due to solar radiation. For the inner surface of MVGSD, the “microclimate” formed by MVGSD near the window significantly reduces the indoor heat gain. In addition, the substrate has a certain heat insulation effect, effectively reducing the temperature transmitted to the inner side of MVGSD and reducing the indoor radiative heat.

(2) $$S=\left(R_{w}-R_{s}\right)/R_{w\;}\times 100\%$$

where S is shading rate, $R_{w}$ is the westward solar radiation, and $R_{p}$ is the substrate surface solar radiation.

## 5. Conclusions

Based on combing the current research results of vertical greening cooling and plant shading, this paper proposes a new plant shading component—MVGSD—which uses plant modules to shade and reduce the heat loss of windows as well as absorb solar radiation. Compared to climbing plant shading, MVGSD has the characteristics of adaptability, adjustability, easy installation, and large-scale production. It uses “trace irrigation” technology to realize the automatic water supply of plants, which is more suitable for high-rise buildings. By comparing the physical and chemical properties (density, porosity, PH, conductivity, water content, etc.) of nine planting substrates, the final planting substrate for MVGSD was determined (i.e., nutrient soil, coir, and perlite in the ratio of 2:3:1), and the following conclusions were drawn through two sets of comparative experiments:

1. The MVGSD effectively reduces the indoor temperature, and the maximum indoor temperature can be reduced by 4 °C.
2. Under photosynthesis, MVGSD with plants can effectively reduce indoor CO_2_ concentration since photosynthesis efficiency is positively correlated with solar radiation. The MVGSD installed in the west has the best photosynthesis effect in the afternoon, and the CO_2_ maximum absorption rate can reach 53.1%. As for the monitoring of CO_2_ concentration, a typical data in a whole day was selected from the monitoring of several consecutive days, although the data of other days are not so obvious. It is difficult to analyze the reasons, which is the limitation of CO_2_ concentration study and the content of our follow-up research. But this result shows that plants can reduce indoor CO_2_ concentration is feasible.
3. The average room temperature installing MVGSD is 2.6 °C lower than the room temperature with a louver, and it also effectively adjusts the indoor humidity in summer, which can be maintained in a suitable and comfortable range.
4. The thermal reaction of MVGSD is different from that of ordinary shading components and has the functions of temperature regulation and self-protection. Plants with good ecological insulation will not convert all absorbed heat to lead temperature rising. Therefore, the internal and external surface temperature of the MVGSD is lower than that of the louver, with a maximum difference of 15.7 °C and 11.26 °C, respectively. The difference between the heat transfer mechanisms of the two shading methods was verified by monitoring and comparing the internal surface temperatures of the windows in the two rooms. MVGSD has a more sophisticated cooling effect in shading than other artificial materials.

In conclusion, MVGSD plays a significant role in cooling and adjusting indoor humidity, which is conducive to improving indoor thermal comfort and reducing the demand for building refrigeration energy consumption in summer. In addition, plants can reduce the indoor CO_2_ concentration under photosynthesis, reducing the demand for fresh air volume for high-rise buildings, thus reducing the energy consumption of the fresh air system. This research provides a new ecological approach to energy saving in building windows and has a certain positive effect on reducing carbon emissions in the building. MVGSD is easy to install, low-cost, and simple to operate, and it can be applied to transparent envelope structures (glass curtain walls, windows, glass domes, etc.) of new buildings to reduce the indoor overheating caused by glass. In addition, for buildings in low-latitude areas, due to the long summers and strong solar radiation, the use of glass curtain walls, windows, and other kinds of glass enclosures in these areas causes a problem with high indoor temperatures, especially for buildings with large window-to-wall ratio. MVGSDs can be used for transparent structural transformation at a low cost for the existing facilities with low comfort. Under the conditions of not damaging the building facade structure, the low-cost installation of MVGSD can effectively alleviate the indoor overheating problem in summer and provide a way to improve the thermal comfort for some low-income groups and low-cost reconstruction projects.

## 6. Patents

This paper’s modular vertical greening shading device has applied for a utility patent (No. ZL 2021 2 2594547.7).

## Figures and Tables

**Table 1:** Proportioning and physicochemical properties of 9 mixed substrates.

No.	Nutrient Soil	Coir	Perlite	Dry Weight (g/cm^3^)	Porosity (%)	Water Content by Capillary (%)
S1	1	1	1	0.148	47.77%	27.53%
S2	1	2	2	0.127	52.86%	23.11%
S3	1	3	3	0.120	50.36%	29.18%
S4	2	1	2	0.169	45.62%	30.77%
S5	2	2	3	0.144	40.75%	27.13%
S6	2	3	1	0.139	46.21%	33.23%
S7	3	1	3	0.179	45.73%	27.30%
S8	3	2	1	0.178	39.77%	28.33%
S9	3	2	2	0.168	50.60%	30.16%

**Table 2:** PH and electrical conductivity of 9 mixed substrates.

No.	PH				Electrical Conductivity (EC)			
	A	B	C	Average	A	B	C	Average
S1	6.6	7.1	7.3	7.0	110.0	118.0	122.0	116.7
S2	5.9	6.1	6.4	6.1	113.0	122.0	109.0	114.7
S3	7.1	7.6	7.8	7.5	109.0	112.0	118.0	113.0
S4	5.8	6.1	6.2	6.0	110.0	112.0	116.0	112.7
S5	5.8	6.0	6.5	6.1	113.0	115.0	124.0	117.3
S6	5.9	6.3	6.4	6.2	242.0	255.0	263.0	253.3
S7	7.5	8.2	8.4	8.0	112.0	119.0	120.0	117.0
S8	5.7	5.9	6.0	5.9	165.0	169.0	181.0	171.7
S9	5.8	5.9	6.1	5.9	114.0	123.0	159.0	132.0

**Table 3:** Comprehensive comparison of 9 mixed substrates.

No.	Dry Weight (g/cm^3^)	Porosity (%)	Water Content by Capillary (%)	PH	EC	Thermal Conductivity (°C/cm)
S1	0.148	47.77%	27.53%	7.0	116.7	1.64
S2	0.127	52.86%	23.11%	6.1	114.7	1.23
S3	0.120	50.36%	29.18%	7.5	113.0	1.68
S4	0.169	45.62%	30.77%	6.0	112.7	1.23
S5	0.144	40.75%	27.13%	6.1	117.3	1.58
S6	0.139	46.21%	33.23%	6.2	253.3	1.91
S7	0.179	45.73%	27.30%	8.0	117.0	1.77
S8	0.178	39.77%	28.33%	5.9	171.7	2.01
S9	0.168	50.60%	30.16%	5.9	132.0	1.79

**Table 4:** Experimental conditions.

Time	Thermal Lab A	Thermal Lab B
1 September 2021	No shade	Window with MVGSD
17 September 2021	Window with Louver	Window with MVGSD

**Table 6:** Comparison of Experiment 1.

	Temperature (°C)			CO_2_ Concentration (ppm)		
	Lab A	Lab B	Difference (A − B)	Lab A	Lab B	Difference (A − B)
MIN	28.7	28.6	0	367	207	−66
MAX	42.4	38.4	4	512	482	245
MEAN	34.34	32.54	1.80	453.76	400.96	52.80

**Table 7:** Comparison of Experiment 2.

	Temperature (°C)			Humidity (RH%)		
	Lab A	Lab B	Difference (A − B)	Lab A	Lab B	Difference (B − A)
MIN	26.60	27.00	−0.40	27.70	43.40	3.30
MAX	40.80	36.90	4.00	59.40	67.30	37.30
MEAN	32.31	31.61	0.70	44.44	54.87	10.42

**Table 8:** Comparison of sunshade.

	Inside Temperature (°C)			Outside Temperature (°C)		
	Louver	MVGSD	Difference (A − B)	Louver	MVGSD	Difference (A − B)
MIN	24.80	24.07	0.66	24.36	24.05	0.28
MAX	48.00	38.55	11.26	52.21	39.79	15.70
MEAN	37.39	31.97	5.43	38.62	32.08	6.54

**Table 9:** Comparison of temperature measuring points in two rooms.

Measuring Points	Thermal Lab A			Thermal Lab B			Maximum Difference
	MIN	MAX	MEAN	MIN	MAX	MEAN	
P1	24.50	49.33	32.79	24.98	34.40	29.60	14.93
P2	23.70	52.04	32.78	23.51	42.47	30.32	10.28
P3	23.63	49.50	32.41	22.58	44.43	29.91	6.45
P4	23.29	56.25	33.09	22.72	45.87	30.53	10.60

## Data Availability

Not applicable.
